# Advances in estimating plasma cells in bone marrow: A comprehensive method review

**DOI:** 10.4102/ajlm.v13i1.2381

**Published:** 2024-07-11

**Authors:** Ethan J. Gantana, Ernest Musekwa, Zivanai C. Chapanduka

**Affiliations:** 1 Department of Pathology, Faculty of Medicine and Health Sciences, Stellenbosch University, Cape Town, South Africa; 2 Department of Haematology, National Health Laboratory Service, Cape Town, South Africa

**Keywords:** plasma cell neoplasms, multiple myeloma, digital pathology, artificial intelligence, haematology, pathology

## Abstract

The quantitation of plasma cells in bone marrow (BM) is crucial for diagnosing and classifying plasma cell neoplasms. Various methods, including Romanowsky-stained BM aspirates (BMA), immunohistochemistry, flow cytometry, and radiological imaging, have been explored. However, challenges such as patchy infiltration and sample haemodilution can impact the reliability of BM plasma cell percentage estimates. Bone marrow plasma cell percentage varies across methods, with immunohistochemically stained biopsies consistently yielding higher values than Romanowsky-stained BMA or flow cytometry alone. CD138 or MUM1 immunohistochemistry and artificial intelligence image analysis on whole-slide images are emerging as promising tools for accurate plasma cell identification and quantification. Radiological imaging, particularly with advanced technologies like dual-energy computed tomography and radiomics, shows potential for multiple myeloma diagnosis, although standardisation remains a challenge. Molecular techniques, such as allele-specific oligonucleotide quantitative polymerase chain reaction and next-generation sequencing, offer insights into clonality and measurable residual disease. While no consensus exists on a gold standard method for BM plasma cell quantitation, CD138-stained biopsies are favoured for accurate estimation and play a pivotal role in diagnosing and assessing multiple myeloma treatment responses. Combining multiple methods, such as BMA, BM biopsy, and flow cytometry, enhances accuracy of diagnosis and classification of plasma cell neoplasms. The quest for a gold standard requires ongoing research and collaboration to refine existing methods. Furthermore, the rise of digital pathology is anticipated to reshape laboratory medicine and the role of pathologists in the digital era.

**What this study adds:**

This article adds a comprehensive review and comparison of different methods for plasma cell estimation in the bone marrow, highlighting their strengths and limitations. The goal is to contribute valuable insights that can guide the selection of optimal techniques for accurate plasma cell estimation.

## Introduction

Plasma cell neoplasms encompass a wide variety of disorders that result from the clonal expansion of terminally differentiated B cells (plasma cells), typically secreting a single homogeneous monoclonal immunoglobulin.^1^ The differentiation between some of these disorders and the monitoring of plasma cell (PC) neoplasms depends on the quantitation of clonal PC in the bone marrow (BM) and the use of biochemical and haematological parameters and imaging techniques.^1,2,3,4^

Quantitation of PC in BM samples has historically relied on manual methods such as microscopy-based cell counting on BM aspirates and immunohistochemically stained trephine biopsies. The surface marker CD138 is sensitive and specific for PC, thus CD138 immunohistochemistry is commonly used for estimating PC in BM biopsies. This practice is supported by the World Health Organization.^1,5,6^ However, various alternative approaches have emerged, each with their strengths and limitations. As the field continues to evolve, it is essential to critically review and evaluate the available methods for estimating PC in BM.

Currently, there is no acceptable reference method for estimating PC in BM, although many methods have been developed and evaluated for accuracy and reproducibility. This method review aims to comprehensively evaluate existing techniques for the quantitation of PC in BM samples. By examining the principles, procedures, advantages, and challenges associated with the various methods, we aim to provide a comprehensive understanding of the options available to clinicians, researchers, pathologists, and haematologists.

In this review the authors examine the factors that affect the reliability of plasma cell estimation and share thoughts on the promising role of new technologies. In addition, the challenges of standardisation, clinical relevance and future directions will stimulate discussion and encourage collaboration in this field. We hope to contribute to the refinement of PC estimation methods and their integration into routine clinical practice.

## Methods

Based on the recommendations by Levac et al., Colquhoun et al. and the methods of Arksey and O’Malley, we conducted a literature search for a narrative (scoping) review article.^7,8,9^ The four main electronic databases (PubMed, Google Scholar, Cochrane Library and Mendeley) were searched to map the existing landscape of methods for estimating bone marrow plasma cell percentage (BMPC%), and to compare and summarise these methods to provide suggestions for the use and further development of appropriate and cost-effective methods for estimating BMPC%, particularly in resource-limited settings. A summary table (Table 1) was created to summarise the data from the literature review and to present the different methods, as well as their advantages and disadvantages, in a clear format. The search was conducted from inception to 31 November 2023 to find relevant articles published in peer-reviewed journals. The search strategy consisted of a combination of keywords and Medical Subject Heading terms related to the topic of interest, such as ‘multiple myeloma’, ‘bone marrow plasma cells’, ‘plasma cell estimation’, etc. Articles that were irrelevant, did not contain a methods section, and were not written in English were excluded. All relevant articles, literature sources and citations were included in the reference list and duplicates were removed.

**TABLE 1:** Summary of various methods used for the estimation of bone marrow plasma cell percentages, highlighting their advantages and disadvantages.

Method	Description	Advantages	Disadvantages
Manual differential count (myelogram)	Microscopic examination of Romanowsky-stained BM slides to count PC as a proportion of all other nucleated cells in a 200–500 cell differential.	Readily available. Direct visualisation of PCs. Easy to perform.	Subjective interpretation. May misrepresent PC count (haemodilution, PC may not ‘fall out’ from BM particles or not aspirated). BMA may underestimate PC% compared to BM biopsies. Unable to determine clonality of PC.
Multiparameter flow cytometry	Uses fluorescently labelled antibodies to identify PC in a BMA sample.	Quantitative (controversial) and qualitative analysis. Differentiate neoplastic PC from normal PC. Determine clonality of PC. Differentiate between MGUS/SMM/MM using an MFC protocol.^29^ Determine risk of MM with MFC and AI-based GBM algorithm.^62^ NGF validated for MRD.	Expensive equipment. Expertise required. PC fragility, haemodilution or clotted samples, processing techniques etc. reduce BMPC%.
Immunohistochemistry	Detection of specific PC antigens (CD138 and MUM1) in tissue sections using chromogenic immunohistochemistry.	Readily available. Identifies specific PC antigens. Most sensitive method for PC estimation compared to BMA. Determine clonality by kappa and lambda antigens. Manual counting methods have been evaluated to reduce subjectivity of assessment.	Requires tissue processing and specialised reagents. Subjective interpretation if overview estimations are performed.
Molecular techniques (ASO-qPCR and NGS)	Based on amplification of unique VDJ rearrangements of *IGHV* gene.	High sensitivity and specificity. NGS does not require a diagnostic specimen. NGS is standardised for general use. NGS validated for MRD.	Requires specialised equipment and expertise. ASO-qPCR requires a diagnostic specimen. Unavailability of NGS in resource-limited settings.
Digital imaging analysis	Computer-based and AI analysis of IHC-stained slides using static images or WSI.	Objective and reproducible results. Guidelines for standardisation and validation available. Open-source AI software available for image analysis. Alternative means (other WSI) to digitise slides and analyse images are available.^57,59^	Initial setup and validation may be time-consuming. WSI scanners are expensive.
Radiology imaging techniques	PET/CT uses a surrogate measure of intracellular glucose metabolism to identify hypermetabolic PC. MRI measure the water and fat composition of tissues.	PET/CT more sensitive for focal lesions. MRI more sensitive for detection of diffuse infiltration. Provide valuable prognostic information.	^18^F-FDG PET/CT cannot reliably predict PC infiltration of < 10%. ^18^F-FDG PET/CT requires hypermetabolically active plasma cells to create a detectable signal. May not replace BM biopsy evaluation.

PC, plasma cell; BM, bone marrow; BMPC%, bone marrow plasma cell percentage; MGUS, monoclonal gammopathy of undetermined significance; SMM, smouldering multiple myeloma; MM, multiple myeloma; MFC, multiparameter flow cytometry; GBM, gradient-boosting machine; NGF, next-generation flow; MRD, measurable residual disease; BMA, bone marrow aspirates; ASO-qPCR, allele-specific oligonucleotide quantitative polymerase chain reaction; NGS, next-generation sequencing; IHC, immunohistochemistry; VDJ, variable, diversity, joining; IGHV, immunoglobulin heavy chain variable; WSI, whole slide imaging; MRI, magnetic resonance imaging; ^18^F-FDG, 18F fluorodeoxyglucose; PET/CT, positron emission tomography/computed tomography; AI, artificial intelligence.

### Pre-analytical factors affecting plasma cell estimation

#### Bone marrow aspiration and processing for accurate visualisation

The first millilitre of aspirated BM represents pure BM elements. The subsequent sampling becomes significantly blood diluted (haemodilution).^10,11^ Ancillary testing including flow cytometry and cytogenetics require additional drawing of BM aspirate (BMA). The first and second draw of BMA yield discrepant results, with the second draw of BMA smears significantly underrepresenting the PC count. This remains important in the evaluation of newly diagnosed multiple myeloma (MM) patients.^12^ Bone marrow aspirate smears are considered the least manipulated sample. Therefore, the numeric discrepancy between the morphological assessment and the flow cytometric assessment cannot be attributed solely to technical or methodological deficiencies. Note that the same degree of discrepancy is not seen with other haematological malignancies such as acute myeloid leukaemia.^13^

Bone marrow aspirate smears are usually stained with Romanowsky-type stains, which are used either in combination or individually. The staining of BM films may be adversely affected by any of the pre-analytical processes.^11^ Plasma cells on Romanowsky-stained BMA are recognised by their distinctive nuclear and cytoplasmic features.^14^

#### Bone marrow trephine biopsy specimens and selection of appropriate antibodies

The common problem with myeloma trephine samples is inadequate or fragmented cores. The biopsy may be inadequate if the same bone puncture site is used for aspiration and biopsy.^15^ Multiple myeloma trephine biopsy should ideally contain five to six intertrabecular spaces that provide a reasonable probability of detecting focal BM lesions.^14^

Bone marrow trephine biopsy specimens are usually fixed in formalin, embedded in paraffin, and stained with haematoxylin and eosin.^11^ Identifying plasma cells on haematoxylin and eosin preparations is relatively easy for the experienced microscopist. However, PC are more easily distinguished from other nucleated cells by immunohistochemistry using antibodies targeting antigens such as CD138 or MUM1. These antibodies are specific to PC in the BM; however, soft tissue tumours such as melanoma, osteosarcoma and osteoblastoma may also stain positive for CD138 on immunohistochemistry.^5,16^

Sections of clotted BMA may be used in cases where it is difficult to obtain a trephine biopsy. The BMA is allowed to clot in a syringe. The clot is then carefully removed, placed in a fixative such as formalin, and processed in the same way as a trephine biopsy, except that no decalcification is required.^11^

### Current methods for estimating plasma cells in bone marrow

#### Manual cell counting on bone marrow aspirate smears

Cellular trails of Romanowsky-stained BM aspirates are used for differential counts under the light microscope, but can be misleading if PC do not ‘fall out’ from the BM particles or are not aspirated due to the patchy nature of MM. Plasma cell are counted as a proportion of all other nucleated haematopoietic cells in a 200–500 cell differential to estimate the percentage of PC in the BM.^11^ Bone marrow aspirate smears often underestimate the percentage of PC compared to BM biopsies, leading to a lack of consensus on the appropriateness of smears for determining the percentage of PC in BM.^17,18,19,20,21,22,23^

In a study conducted in the United States in 2007, Al-Quran et al. evaluated CD138-stained BM specimens and showed poor intraobserver concordance with an intraclass correlation coefficient of 0.55 for one observer and an intraclass correlation coefficient of 0.47 for another between PC quantitation on BM aspirate smears and CD138 immunohistochemistry.^19^ Smith and Elnawawi, in a study conducted in the United States in 2008, found that aspirate smear PC counts were considerably lower (mean 10.6; standard deviation [s.d.] ± 13.1) than the counts by other methods such as overview estimates (mean 19.2; s.d. ± 20.4) and manual counts on CD138 immunohistochemistry (mean 23.1; s.d. ± 17.7), and attributed this to an under-representation of PC in aspirate smears.^18^

Despite this percentage discrepancy, one study found a significant linear correlation between BMPC% in aspirate enumeration and CD138 immunohistochemistry (*r* = 0.71 [Pearson correlation coefficient]), which suggests the possibility of extrapolating the true BMPC% from the manual count on BMA smears.^23^ Morphological assessment of BMA smears has limitations, including the assessment of tumour burden and the determination of PC clonality. It is therefore best used in combination with immunohistochemistry and with or without multiparameter flow cytometry (MFC) when detection of clonality with immunohistochemistry is difficult, especially in the context of post-therapeutic hypocellular BM samples or a low tumour burden.^1,3,4,14,24^ The World Health Organization allows the BMPC% to be determined either by manual counting of the aspirate or by CD138 immunohistochemistry. It is recommended to consider the highest PC percentage of aspirate or trephine if there is a discrepancy between the two methods.^1,3^

#### Multiparameter flow cytometry on bone marrow aspirate specimens

The literature supports the use of MFC in the diagnosis and follow-up of MM, particularly regarding establishing the clonal nature of the PC.^25,26,27^ Multiparameter flow cytometry has been shown to provide more prognostic information for overall survival than morphological assessment in patients with MM.^25^

The use of MFC to quantify PC in BM is more controversial. It has been shown that the percentage yield of PC is consistently lower with flow cytometric methods than with morphological assessment of smears from BMA.^12,13,22,25,27,28,29^ This discrepancy is thought to be because of PC fragility, differences in sample quality, dilution with peripheral blood (haemodilution) or clotted specimens and cellular processing techniques used in flow cytometry that reduce the PC count.

In contrast to this, a 2017 study by Matsue et al. in Japan showed that the BMPC% on aspirate smear counts correlated with flow cytometry (*r* = 0.93), but BMA smear and flow cytometry significantly underestimated the PC percentage compared to BM biopsy or clot stained with CD138 immunohistochemistry.^30^ Another study showed that even though a discrepancy was seen with morphological enumeration average (60%) in MM patients compared to flow cytometry (20%), this discrepancy was not found between morphological and flow cytometry assessment in acute myeloid leukaemia samples.^13^ Based on these findings, the discrepancy regarding MM cannot be attributed solely to technical or methodological problems. It may stem from unique interaction between malignant PC and the BM niche they occupy, including bone surfaces, basement membranes, and lipid-enriched spicules.^13^ Furthermore, patchy marrow infiltration, hypoplastic or fibrotic marrows may cause the underestimation of PC percentages.

With the concern of the significant discrepancy between morphological assessment and flow cytometry PC percentage estimation, Frebet et al., in a study in France in 2011, developed an MFC protocol that uses specific antibody combinations to include PC (CD138+/CD38+) and haematopoietic precursors (CD45+/CD117+/CD34+), and exclude erythroblasts and debris (CD36+/CD45–), to calculate a plasma cell:precursor ratio.^29^ A plasma cell:precursor ratio threshold of 2 was validated as a discriminative tool to differentiate between monoclonal gammopathy of undetermined significance and smouldering MM with high specificity (84%) and sensitivity (81%).^29^

#### Immunohistochemistry on bone marrow biopsy trephine specimens

CD138 (Syndecan-1) is a cell surface, sulphate-rich proteoglycan adhesion molecule which is expressed in the late stages of B-cell differentiation and on PC. CD138 is a highly sensitive and specific marker for BMPCs, making it useful in identification and enumeration of BMPC.^5,6^ CD138 staining has the advantage of improved PC identification compared to haematoxylin and eosin-stained sections. The estimation of PC in BM biopsies using CD138 immunohistochemistry, as suggested by the World Health Organization, is generally performed using a semi-quantitative overview estimation method.^1^

In their 2008 study, Smith and Elnawawi proposed an alternative to overview estimation by performing a manual count on a single selected field of view at high magnification (40x objective). The manual count (mean 23.1; s.d. ± 17.7) correlated better with computer-assisted image cytometry (mean 21.4; ± 10.7 s.d.) than the overview estimates (mean 19.2; s.d. ± 20.4), on 44 CD138-stained core biopsy specimens.^18^ Importantly, the s.d. of selected field counts in this study and the overview estimation are high, and may be clinically relevant for the diagnosis and evaluation of treatment response of PC neoplasms. A single area was selected for the manual counting method of PC estimation, even though MM is a patchy disease and shows considerable variation in BM infiltration.

In their 2017 study in Japan, Matsue et al. counted more cells (500–2500) in 2–5 representative microscopic fields at low magnification in CD138-stained BM clot and biopsy sections. They showed significant correlation between the marrow clot and biopsy sections (*r* = 0.96); however, quantification by BMA smear (BMPC% = 3.7 [interquartile range {IQR}: 1.2–10]) and flow cytometry (odds ratio = 2.4 [IQR: 0.8–6.2]) underestimated PC percentage compared to BM biopsy (odds ratio = 13.3 [IQR: 6.7–36.2]) and BMA clot (odds ratio = 12.8 [IQR: 6.8–31.9]).^30^ Samples with a nodular or diffuse pattern of the PC and cells with Golgi staining or non-specific cytoplasmic staining were excluded from the study.

In their 2023 publication in South Africa, Gantana et al. evaluated a counting method in which reviewers selected three representative biopsy areas with low, intermediate, and high PC densities, followed by a manual count in each of these areas and averaging the BMPC% for each sample. This study showed superior interobserver concordance with the manual counting method at low PC tumour burden compared to an overview estimation method (intraclass correlation coefficient = 0.105).^31^

Many studies show significantly higher numbers of BMPC% on CD138-stained immunohistochemistry biopsies compared to BMA.^12,18,19,21^ Suggested reasons for this discrepancy are the patchy nature of MM, haemodilute BMA, and poor sample quality. For these reasons, CD138 immunohistochemistry is regarded as the most sensitive method for PC estimation.

Immunohistochemistry determination of PC percentage is valuable for diagnosis and monitoring treatment response, notably the complete and stringent complete response criteria as defined by the International Myeloma Working Group (IMWG).^3^ Furthermore, BM trephine PC percentages have shown to correlate with corresponding end organ disorders, quantitative fluorescence in situ hybridisation results, and overall survival in patients with a low BMA PC percentage.^24^

#### Molecular techniques

Molecular techniques are based on the amplification of unique variable, diversity, joining rearrangements of the immunoglobulin heavy chain variable (*IGHV*) gene on chromosome 14, which are specific for each PC clone and can be used for identification by amplification of the variable, diversity, joining sequence. The light chain genes lack the D segment, which reduces the sensitivity of detecting the PC clone.^32^ Patient-specific *IGHV* gene rearrangements are amplified and sequenced from *IGHV* gene-specific complementary DNA to determine the clone-specific variable gene sequence.^32,33^ This should be done at diagnosis, to design primers for real-time polymerase chain reaction (PCR) quantification for determination of the level of tumour contamination in a sample after chemoimmunotherapy and stem-cell transplants to be used for follow-up monitoring. The sense primers are usually derived from the second complementarity-determining region and the antisense primer from the highly hypervariable third complementarity-determining region in the *IGHV* gene to be used for the amplification of the sequence of interest during follow-up.^34,35,36^

Molecular techniques such as allele-specific oligonucleotide quantitative PCR (ASO-qPCR) and next-generation sequencing (NGS) are used during follow-up for the quantification of clonal plasma cells to evaluate treatment response and detect measurable residual disease (MRD) rather than quantification at diagnosis.^4^ The use of ASO-qPCR for the detection of these specific variable, diversity, joining sequences correlates well with MFC in MRD quantitation (*r* = 0.881, *p* < 0.001), but its application was limited to 42% of cases in the study due to lack of clonality detection, unsuccessful sequencing and suboptimal ASO performance.^37^ This complicates the practicality of using only molecular techniques for this indication.

#### Radiological imaging techniques

Radiological imaging plays a significant role in the diagnosis and management of MM as approximately 90% of MM patients develop bone disease.^38^ Radiological imaging may suggest diagnosis, assess possible bone complications, and determine response to treatment or disease progression.^1,4,39^

Radiographic examination (skeletal survey) to identify lytic bone lesions has been replaced by more sensitive alternatives such as whole body, low-dose computed tomography (CT), 18F fluorodeoxyglucose (^18^F-FDG) positron emission tomography/CT (PET/CT) scanning, and magnetic resonance imaging (MRI). In under-resourced countries, skeletal surveys are still widely used due to the lack of availability of superior modalities especially for detection of osteolytic lesions in the spine and pelvis.^40,41,42,43,44^ Positron emission tomography/CT and MRI can be used to assess tumour burden and disease activity.^45,46^ Positron emission tomography/CT uses a surrogate measure of intracellular glucose metabolism to distinguish between metabolically active and inactive sites of proliferating cells, while MRI examines the water and fat composition of tissues.^47^ Magnetic resonance imaging is therefore more sensitive for the detection of diffuse infiltration and PET/CT is better for the assessment of focal lesions, especially when these lesions are outside the field of view of MRI.^44^ The patchy and focal nature of MM infiltration means that disease burden measured on a random BM biopsy may not be representative. Furthermore, seven focal lesions on spinal MRI and more than three lesions and extramedullary disease on PET/CT have been shown to be prognostically significant, and it is therefore recommended that the absolute number of focal lesions should be reported.^39,47,48,49,50^

A South African study in patients at a tertiary hospital showed good concordance between representative BM biopsy samples and ^18^F-FDG PET/CT image analysis. However, this did not reliably predict PC infiltration of < 10% or diffuse non-hypermetabolic marrow involvement on imaging.^51^ This study further emphasised the patchy nature of the disease where patients with irregular BM infiltration may result in the underestimation of PC infiltration.^51^ Based on these results, the use of PET/CT imaging can avoid the need for repeated BM biopsies in most patients with diffuse and irregular BM uptake, but cannot replace BM biopsy to determine BMPC%. Imaging will continue to play a role in assessing myeloma-defining events, providing prognostic information, and evaluating response to treatment by providing complementary information.^4,39^

### Emerging technologies and innovations

#### Digital pathology

Digital pathology, with or without incorporation of artificial intelligence (AI), is growing in popularity while presenting both challenges and opportunities for the future. Careful consideration is required when introducing this new area of medicine to patient care. Digitisation of immunostained BM biopsy sections can be done by taking still images with a microscope camera or dynamic images with whole slide imaging (WSI) systems. Images can be analysed manually or using AI. Herein, we review several studies that have explored the use of these innovations in estimating BMPC%.

Computer-assisted image analysis (CIA) for estimating BMPCs in BM biopsies dates to the early 2000s. Computer-assisted image analysis was shown to be superior to simple visual estimation of CD138 immunohistochemistry, due to elimination of subjectivity. In a Swiss study by Went et al. in 2006, CIA was performed using image processing software (KS 300, Carl Zeiss AG, Feldbach, Switzerland) after digitising selected fields with a light microscope and colour video camera.^52^ This study showed minimal interobserver and intraobserver variability (determined by Spearman’s rank correlation coefficient) between two reviewers using CIA.^49^ A study by Stifter et al. in Croatia in 2010 used similar image processing software (Alphelys Spot Browser 2.4.4., Plaisir, France) for CIA after digitisation using a similar method showed high reproducibility.^24^ The CIA evaluated the relative area of overall positive staining rather than a calculated percentage of individually positively stained cells. Computer-assisted image analysis is therefore significantly influenced by background staining, large atypical PC, and PC clusters.^21,49^

Technology has improved so that an entire biopsy slide can be digitised with WSI scanners, and more advanced software programmes than those used in CIA and AI are used to analyse the images.^50,51,53^ The WSI, used together with an image analysis tool and established algorithms (Asperio; Positive Pixel Count v9, Vista, California, United States), has shown good correlation with visual estimates and counts by experienced pathologists. However, it is often influenced by the intensity of the staining, leading to an overestimation of BMPC%.^50^ The overestimation is due to a ‘halo’ effect in cases with strong positivity, but in these cases WSI still showed good correlation (*r* = 0.857) with visual estimates.^53^ Another study also claimed that automated digital enumeration of the entire, immunohistochemically stained (CD138 and MUM1) biopsy can accurately determine PC burden, irrespective of pattern or extent of disease.^54^ It also showed that manual visual assessment (a 500-cell manual count in ‘representative regions’) tends to overestimate PC burden.^54^

The use of an open-source digital image analysis tool, QuPath, was validated by Baranova et al., in a study published in Canada in 2021, for the automation of PC quantification in BM biopsies from patients with MM.^55^ They accurately and reliably estimated BMPC% on CD138-stained slides with good correlation between manual counting and software image analysis (Pearson’s *r* = 0.96, *p* < 0.001).^56^ The study used an Aperio ScanScope XT scanner (Aperio Technologies, California, United States) for digitisation of the biopsies to generate WSI for analysis.^56^ Similar studies were done by Fu et al. in Canada and the United States in 2022, and by Lomas et al. in the United Kingdom in 2022, using WSI.^57,58^

Whole slide imaging may be used to overcome the considerable variability in cellularity and distribution of plasma cells in BM biopsies by providing images of the full length of the biopsy. Whole slide imaging scanners are not readily available in resource-limited settings, hence alternative means of digitising and analysing biopsies are still required. Using the AI of QuPath, Gantana et al., in their South African study published in 2023, counted CD138-positive PC in BM biopsies and showed that digital analysis is superior to both a manual counting method and a previously reported overview estimation method, regardless of the tumour burden of the sample.^59^ In this study, digitisation of representative biopsy areas with low, intermediate and high PC densities was done with a standard microscope and camera rather than WSI. Fu et al. developed a web application that utilises their developed convolutional neural network, which allows pathologists to upload single images from microscope cameras to obtain a percentage of plasma cells in real time based on the CD138-stained plasma cells on that image.^57^ These methods may be more accessible to low-resource settings than using WSI.

The advantage of digital image analysis is therefore considerable. It may be incorporated into routine use, but slides need to be of equal quality. This requires standardisation of biopsy processing protocols.^52^ One study showed that all pre-analytical aspects affect the appearance of tissue sections and their suitability for digital pathology and therefore recommended visually checking the PC estimates and adjusting both parameters and thresholds accordingly.^60^ The College of American Pathologists recently released an updated guideline for the validation of WSI for diagnostic purposes.^61^

#### Artificial intelligence

The accurate determination of BMPCs plays a central role in the diagnosis, prognosis, the choice of therapy and the monitoring of PC neoplasms and is therefore essential. Artificial intelligence, in combination with methods such as flow cytometry and NGS, can provide information to predict risk status and potentially change the diagnostic criteria and risk stratification for MM.

In their 2022 publication in France, Clichet et al. used an AI-based gradient-boosting machine algorithm to develop a decision tree for determining the risk of MM in a cohort of PC neoplasms. They used MFC (10-colour single tube) parameters including PC %, pathological to normal PC ratio, total PC to haematogone ratio, total PC to progenitor CD117+ ratio, mean fluorescence intensity of CD38 and CD27 from pathological PC, and mean fluorescence intensity of CD27 from normal cells at diagnosis. The AI-based decision tree showed a sensitivity of 94.2%, a specificity of 93.7% and an independent validation success rate of 91.0% without misclassification between monoclonal gammopathy of undetermined significance and smouldering multiple myeloma.^62^ This online diagnostic tool is currently freely available and could be used for cases with diagnostic dilemmas in PC neoplasms.

Yenamandra et al., in their study published in the United States in 2021, used data at the time of diagnosis which included variables such as patient age, white blood cell count, percentage of PC in the BM on the myelogram, high-risk cytogenetic alterations, and NGS. These data were used to create neural networks with which they developed a predictor for the risk status of MM, which has the potential to predict high-risk patients.^63^ The limitation is lack of estimated BMPC% in the BM biopsy sections.

With picture archiving and communications systems as the standard in many tertiary institutions, radiology has a significant potential for radiomics, the use of pattern recognition for the extraction of quantitative descriptors from imaging data.^64^ Radiomics and computational algorithms may potentially be used for quantitative imaging in MM patients by assessing different bone lesions in PET/CT imaging and MRI.^65^ This is an interesting and dynamic field, in early phase of development.^65,66^ However, this depends on the availability of technology in some tertiary facilities with PET/CT and MRI technologies.

### Estimation of plasma cells in bone marrow for measurable residual disease

There are several strategies for MRD detection in myeloma, including MFC, qPCR, and high-throughput sequencing. The two currently validated MRD methods recommended by the IMWG are next-generation flow cytometry and NGS. Next-generation flow refers to the 8-colour 2-tube flow cytometry method developed by the EuroFlow consortium, which uses specific combinations of antibodies to identify and cross-reference neoplastic PC. It has a sensitivity of 10^-6^. In contrast, conventional 4–8 colour flow cytometry methods can only detect neoplastic PC with a sensitivity of 10^-4^.^67,68^

#### Next-generation flow cytometry

Multiparameter flow cytometry is a sensitive and rapid approach to assessing treatment efficacy with confirmed prognostic value that predicts progression-free and overall survival independent of categorical response assessment and patient biology.^4,37,68,69,70^ The IMWG defined ‘flow MRD negative’ as the absence of phenotypically aberrant clonal PC detected by BM next-generation flow cytometry with a minimum sensitivity of 1 in 10^5^ nucleated cells. The same minimum sensitivity is proposed for the ‘sequencing MRD negative’ state detected by NGS.^4^

The threshold used to distinguish between MRD positive and MRD negative may vary from sample to sample, and may change as both technologies and treatments improve. The International Clinical Cytometry Society proposed a consensus guideline for the use of flow cytometry for MRD. The International Clinical Cytometry Society recommends the use of at least five initial gating parameters (CD38, CD138, CD45, forward scatter and side scatter) within the same aliquot to accurately identify the total PC compartment and to specify the limit of detection, with a limit of detection of 0.001% (requiring a total number of 3 × 10^6^ cells for analysis) and ideally a limit of quantification of 0.001% (requiring a total number of 5 × 10^6^ cells for analysis).^67^ The International Clinical Cytometry Society recommendation is to report limit of detection and limit of quantification for each case based on the number of events below the detection limit and the number of events.^71^ Furthermore, CD9, CD56, CD27, CD81 and CD117 should be analysed in combination with the initial gating parameters for detection of MRD in MM.^72^ Another method, the Memorial Sloan Kettering Cancer Centre 10-colour single tube method, was compared with the IMWG-recommended EuroFlow 8-colour 2-tube method and showed a high degree of agreement (*r*^2^ = 0.97) with the residual disease burden detected with abnormal PC.^73^

It is noteworthy that a BM aspirate sample containing 10 x 10^6^ cells is required for consistent disease detection, rapid processing avoids cell degeneration, and the use of therapeutic monoclonal antibodies such as daratumumab can lead to false-negative MRD results. In addition to sample processing problems, immunophenotype in MM has been shown to be unstable, and many patients (41%) change immunophenotype, which can complicate interpretation.^74^

In an attempt to overcome the potential loss of typical gating markers (e.g., CD38 and CD138) after treatment with monoclonal antibodies, the members of the signalling lymphocyte activation molecules family, CD229 and CD319, have been identified as new gating markers for PC in flow cytometric immunophenotyping, particularly in relapsed or resistant cases of MM.^75,76,77^ The CD229 marker has a strong and homogenous expression in PC while CD138 expression showed significant variability.^75,76^ The new markers may prove to be useful additions for gating clonal PC, estimating PC in BM and assessing MRD in relapsed and resistant MM. It makes sense for pathologists to familiarise themselves with these novel markers and MRD methods as monoclonal antibody therapies such as anti-CD38 become more readily available in resource-limited settings.

#### Next-generation sequencing

The use of NGS, as with ASO-qPCR, is based on the identification and amplification of the sequence of interest, usually in the variable regions of the IGH gene on chromosome 14. However, for the follow-up of patients using PCR-based methods, a patient-specific primer must be developed from the diagnostic sequence. With NGS, these regions are amplified and sequenced with specific primers that are not patient specific.^36^ For this reason, among others, ASO-PCR has been largely replaced by NGS methods because patient specific probes are not as reliable in identifying clonal IGH after somatic hypermutation compared to NGS during follow-up.^36,37,78,79^ This is an indication of the superior applicability of NGS over the need to develop patient-specific primers. Next-generation sequencing is not entirely free from the effects of somatic hypermutation as it uses consensus primers for clonality detection and subsequent MRD analysis. Cost and poor availability are disadvantages of NGS, especially in resource-limited settings. In addition, due to the labour-intensive nature and difficulties in interpreting the results of NGS, a high level of expertise is required. But it remains a useful instrument because it is very sensitive and has been sufficiently standardised for general use.

### Summary of reviewed methods for plasma cell estimation

The quantitation of BMPC% on Romanowsky-stained BMA alone may misrepresent the accurate count of PC due to the patchy nature of infiltration and sample haemodilution. Bone marrow PC percentage is consistently lower on BMA as compared to immunohistochemistry-stained BM biopsies. Flow cytometry alone yields consistently lower results compared to BMA myelograms and are not readily available in resource-constrained settings. Flow cytometry is, however, useful for determination of PC clonality, MRD, and risk stratification of MM. A combination of BMA, BM biopsy, and flow cytometry assessment of PC yield superior results compared to using each sample on its own.

CD138 or MUM1 immunohistochemistry improves PC identification compared to haematoxylin and eosin and other stains. Manual counting in representative areas of CD138-stained biopsies has been shown to improve reproducibility, compared to overview estimation. Use of AI image analysis on WSI or static images may currently be the best method for BMPC estimation. Guidelines for the standardisation and validation of digital techniques have been published and using static images in combination with open-source software for image analysis will be possible in situations with limited resources.

Radiological imaging is crucial for the diagnosis and treatment of MM. Introduction of new technologies such as double-energy CT and radiomics as standard practice may necessitate changes to IMWG guidelines. These techniques are still in the early stages of development for the assessment of patients with MM and are an exciting and rapidly evolving field. Further development will be required before radiological imaging can replace BM biopsy for BMPC quantitation.

Allele-specific oligonucleotide-qPCR and NGS are commonly used to quantify clonal PC in the follow-up of treatment response and detection of MRD. Allele-specific oligonucleotide-qPCR correlates well with MFC for MRD quantification, but has limitations in clonality detection and sequencing, which affects its applicability. Next-generation sequencing has replaced ASO-qPCR because it is more sensitive and does not require patient-specific primers for MRD monitoring, but using either molecular technique for this purpose can be challenging.

## Conclusion

Diagnosis and classification of PC neoplasms depends on accurate quantification of PC in BM biopsy. Although there is no consensus on the optimal method for estimating PC in BM, CD138-stained biopsies are the preferred sample for this estimation and remain invaluable for diagnosis and assessment of response. No sample or method should be used in isolation, as they are better used in combination for this estimation, and laboratories without advanced techniques should continue to use the best methods available to them. In our quest for a gold standard method, this will require continued research and collaboration to expand and improve the currently published methods. Finally, digital pathology is likely to redefine the way we practise laboratory medicine and the role of the pathologist in this new digital age, and limited resources need not necessarily be a limiting factor.
